# Supplementary material for: Evaluating the Effect of Iron(III) in the Preparation of a Conductive Porous Composite Using a Biomass Waste-Based Starch Template
Source: Polymers (Basel). 2023 Jun 2;15(11):2560. doi: 10.3390/polym15112560 (PMC10255400; doi:10.3390/polym15112560)
Supplement: Supplementary file 1 [file polymers-15-02560-s001.zip › polymers-2291667-supplementary.pdf]

Supporting Information:

# Evaluating the effect of iron(III) in the preparation of a conductive porous composite using a biomass waste-based starch template

**Laria Rodríguez-Quesada[1*], Karla Ramírez-Sánchez[2], Sebastián León-Carvajal[1], Giovanni Sáenz-Arce[3-4], Fabián Vásquez-Sancho[5-6], Esteban Avendaño-Soto[5-6], Juan José Montero-Rodríguez[7] and Ricardo Starbird-Perez[2*]**

1 Master Program in Medical Devices Engineering, Instituto Tecnológico de Costa Rica, Cartago 159-7050, Costa Rica.
2 Centro de Investigación en Servicios Químicos y Microbiológicos (CEQIATEC), Escuela de Química, Instituto Tecnológico de Costa Rica, Cartago 159-7050, Costa Rica
3 Departamento de Física, Facultad de Ciencias Exactas y Naturales, Universidad Nacional, Heredia 86-3000, Costa Rica
4 Centro de Investigación en Óptica y Nanofísica, Departamento de Física, Universidad de Murcia, 30100 Murcia, Spain
5 Materials Research Science and Engineering Center (CICIMA), University of Costa Rica, San José 11501-2060, Costa Rica
6 School of Physics, University of Costa Rica, San José 11501-2060, Costa Rica
7 Escuela de Ingeniería Electrónica, Instituto Tecnológico de Costa Rica, Cartago 159-7050, Costa Rica;
* Correspondence: larodriguez@itcr.ac.cr (L.R.-Q.); rstarbird@itcr.ac.cr (R.S.-P.)

**Contents:**

**Table S1** Physical properties of the porous obtained materials at each stage of synthesis.

**Table S2** Physisorption Nitrogen physisorption data of cryogel template and conductive porous composites at different soaking times.

**Table S3** Elemental composition of porous structures synthesized with PEDOT at different immersion times in iron(III) solution.

**Table S4.** Fitting of electrochemical impedance spectroscopy data using the R(RQ)(RQ) circuit model.

**Figure S1.** Starch template chemical and electrical measurements: elemental mapping (a) SEM imagen, (b) carbon, (c) oxygen (d) energy-dispersive spectroscopy spectra and (e) Nyquist plot (inset shows cyclic voltammetry (CV) curve).

**Figure S2.** Infrared spectroscopy of the different samples polymerized after being soaked for (a) 0.5, (b) 8, (c) 24, and (d) 48 hours in an iron(III) solution.

**Table S1. Physical properties of the porous obtained materials at each stage of synthesis.**

| Sample | $\varrho_{skel}$ g·cm$^{-3}$ | $\varrho_{bulk}$ g·cm$^{-3}$ | ε % | Vp |
|---|---|---|---|---|
| St | 1.470 ± 0.010 | 0.140 ± 0.010 | 90.476 ± 5.849 | 6.460 ± 0.417 |
| St/iron(III) | 1.500 ± 0.006 | 0.195 ± 0.003 | 87.000 ± 1.382 | 4.460 ± 0.070 |
| St/iron(III)/PEDOT | 1.337 ± 0.008 | 0.201 ± 0.010 | 84.966 ± 4.257 | 4.227 ± 0.211 |

**Table S2.** Physisorption Nitrogen physisorption data of cryogel template and conductive porous composites at different soaking times.

| Sample | Vp Cc·g$^{-1}$ | Pore Radius nm | Surface area m$^2$·g$^{-1}$ |
|---|---|---|---|
| St- 0 h | 0.003 | 2.01 | 6.899 |
| PEDOT-St 0.5 h | 0.004 | 2.01 | 5.096 |
| PEDOT-St 8 h | 0.002 | 2.02 | 0.357 |
| PEDOT-St 24 h | 0.002 | 1.63 | 0.225 |
| PEDOT-St 48 h | 0.000 | 1.17 | 0.316 |

**Table S3.** Elemental composition of porous structures synthesized with PEDOT at different immersion times in iron(III) solution.

| Soaking time in iron(III) solution (hours) | Element (%) | | | |
|---|---|---|---|---|
| | N | C | H | S |
| 0.5 | 0.06 | 43.09 | 5.61 | 10.28 |
| 8 | 0.06 | 47.20 | 4.45 | 18.07 |
| 24 | 0.04 | 44.81 | 4.76 | 14.86 |
| 48 | 0.06 | 41.65 | 5.49 | 9.70 |

**Table S4.** Fitting of electrochemical impedance spectroscopy data using the R(RQ)(RQ) circuit model.

| Circuit element | Immersion time in iron(III) (h) | | | |
|---|---|---|---|---|
| | 0.5 | 8 | 24 | 48 |
| Rs1 (Ω) | 2502.2 | 31137 | 9.4186E$^{5}$ | 1.5582E$^{5}$ |
| Rp1 (Ω) | 3949.7 | 1.482E$^{7}$ | 3.5438E$^{7}$ | 1.993E$^{5}$ |
| CPE1 (F) | 5.636E$^{-12}$ | 1.125E$^{-10}$ | 1.277E$^{-10}$ | 6.516E$^{-10}$ |
| CPE1.N | 0.84 | 0.83731 | 0.52077 | 0.82964 |
| Rs2 (Ω) | - | - | 1355.9 | 2627.4 |
| Rp2 (Ω) | - | - | 5.2739E$^{6}$ | 1.9695E$^{5}$ |
| CPE2 (F) | - | - | 5.8335E$^{-12}$ | 1.0885E$^{-11}$ |
| CPE2.N | - | - | 0.92928 | 0.81716 |

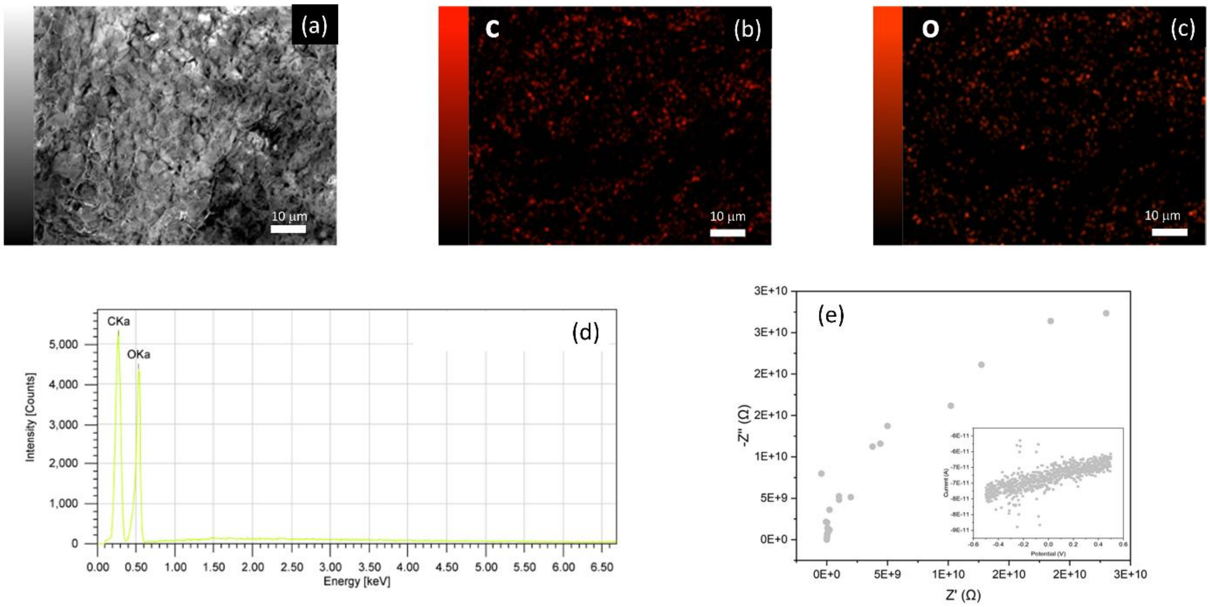


**Figure S1.** Starch template chemical and electrical measurements: elemental mapping (a) SEM imagen, (b) carbon, (c) oxygen (d) energy-dispersive spectroscopy spectra and (e) Nyquist plot (inset shows cyclic voltammetry curve).

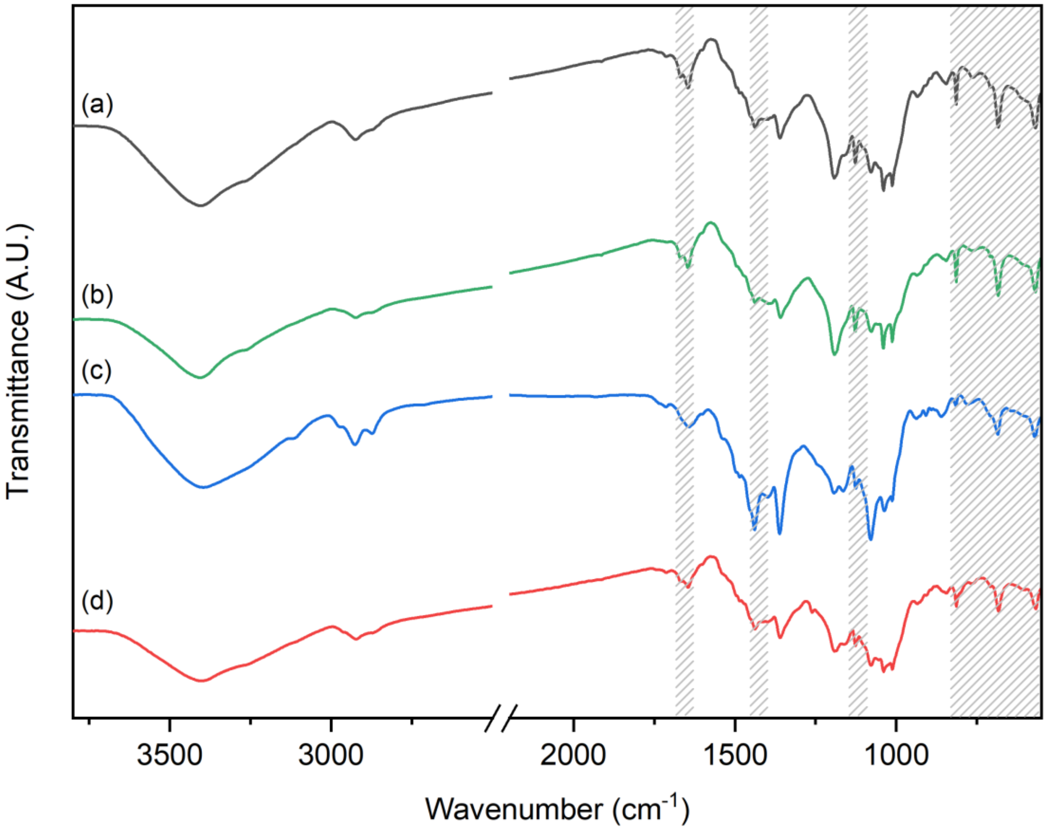

**Figure S2**. Infrared spectroscopy of the different samples polymerized after being soaked for (a) 0.5, (b) 8, (c) 24, and (d) 48 hours in an iron(III) solution.
